# Fabrication and Application of Turmeric Extract-Incorporated Oleogels Structured with Xanthan Gum and Soy Lecithin by Emulsion Template

**DOI:** 10.3390/gels10010084

**Published:** 2024-01-22

**Authors:** Su Jung Hong, Gye Hwa Shin, Jun Tae Kim

**Affiliations:** 1 Department of Food and Nutrition, Kyung Hee University, Seoul 02447, Republic of Korea; tnwjd0799@naver.com; 2 Department of Food and Nutrition, Kunsan National University, Gunsan 54150, Republic of Korea; 3 BioNanocomposite Research Center, Kyung Hee University, Seoul 02447, Republic of Korea

**Keywords:** emulsion template, turmeric extract, oleogels, xanthan gum, soybean lecithin

## Abstract

Turmeric extract (TE)-loaded oleogels (TE-OG) was fabricated by an emulsion template technique using xanthan gum (XG) and soy lecithin (SL) as oleogelators. The formulation for TE-OG was optimized using 0.32% XG, 1.2% SL, and 1.0% TE. The optimized TE-OG had a minimal particle size of 810.23 ± 10.68 nm as measured by the dynamic light scattering (DLS) method, and a high encapsulation efficiency (EE) of 96.62 ± 0.56%. Additionally, the optimized TE-OG exhibited a favorable zeta potential of -27.73 ± 0.44 mV, indicating the good stability of the TE-OG due to the electrostatic repulsion between particles. TE-OG formulated with 0.32% XG and 1.2% SL was subjected to frequency sweep testing to evaluate its solid-like rheological behavior. The oil-binding capacity (OBC) of TE-OG was consistently maintained above 99.99%. In vitro digestion of TE-OG demonstrated the potential of the emulsion template for controlled release, with less than 20% of the encapsulated curcumin being released in simulated gastric fluid (SGF), whereas nearly 70% was released in the simulated intestinal fluid (SIF). Moreover, TE-OG affected the rapid release of free fatty acids (FFAs), which have a positive effect on the digestion of triacylglycerols found in soybean oil (SO). TE-OG was further used as an alternative to commercial butter to produce pound cakes, and their rheological properties were compared to those of the pound cake prepared using commercial butter. The pound cake prepared using TE-OG showed a noticeable decrease in hardness from 10.08 ± 1.39 N to 7.88 ± 0.68 N and increased porosity, demonstrating the inherent capability of TE-OG to enhance the overall quality standards of bakery products.

## 1. Introduction

The rapidly growing consumer demand for healthier dietary choices is evident in the food industry [1]. In particular, solid fat has a high saturated fat content, so it is recognized as a potential cause of chronic diseases such as diabetes [2], obesity [3], cardiovascular disease [4], and even cognitive disorders [5] with excessive consumption. The World Health Organization (WHO) recommends limiting daily saturated fat intake to less than 10% of total calorie intake and switching from saturated solid fat intake to unsaturated liquid oil intake [6]. As a result, a variety of solid fat substitutes have been developed utilizing lipids, carbohydrates, and proteins for a variety of food applications. Despite advances, these substitutes still face challenges in adequately replicating comprehensive aspects such as the texture, processability, and ease of handling of solid fats [7]. The ongoing challenge is bridging the gap between changing health-conscious consumer preferences and developing solid fat alternatives that integrate seamlessly into a variety of food processing applications.

A promising solution of interest is vegetable oil-based oleogelation, which is a technology that gelates liquid oil to give it properties similar to a solid fat. Oleogelator, with its ability to create a thermally reversible gel matrix with a three-dimensional structure, plays a pivotal role in this process [8]. Generally, the methods used to prepare oleogels (OGs) can be categorized into two approaches: the direct method and the indirect method. The direct method is the most common method and involves dispersing hydrophobic oleogelators such as waxes [9] or monoacylglycerides [10] into oil at high temperatures. However, it still presents some problems, including containing a certain amount of saturated fat and having an undesirable waxy taste.

Another approach worth exploring is polymer gelation, which takes advantage of the commercial availability and cost-effectiveness of polymers [11]. However, due to their hydrophilic nature, polymers require indirect methods, such as an emulsion template approach to structure hydrophobic oils [12]. This multi-step process involves preparing an oil-in-water (O/W) emulsion using structuring agents, followed by dehydration and homogenization. Here, polysaccharides act as thickening agents, increasing the viscosity of the aqueous phase and contributing to the long-term physical stability of O/W emulsions [13]. To obtain kinetically stable emulsion-templated OGs, it is essential to incorporate molecules with surface-active properties that can disperse and stabilize the oil droplets in the initial formulation. Xanthan gum (XG), an anionic polysaccharide, is widely used in the food emulsion field, where it is known to increase viscosity and improve emulsion stability [14]. Combining XG with amphoteric carbohydrates or proteins has shown promise in stabilizing O/W emulsions for OG production [11]. In particular, recent studies have investigated the synergistic potential of combining XG with proteins such as gelatin or egg protein to achieve promising results in OG development [15,16,17]. Soy lecithin (SL) is a representative ionic emulsifier widely used in the food industry. The use of SL does not raise any regulatory issues within the field of food law and adds nutritional value, mainly due to its high content of polyunsaturated fatty acids [18].

In recent developments, OG has emerged as an effective tool to influence lipid digestion and promote the delivery of nutrients or bioactive molecules [19,20,21,22,23]. Chuesiang et al. (2022) [19] investigated the effects of OG, which directly disperses curcumin and OG based on a curcumin-loaded nanostructured lipid carrier (Cur-NLC), on the encapsulation efficiency and intestinal absorption of curcumin. During in vitro gastrointestinal digestion, Cur-NLC-based OGs enhanced the release of FFAs, while high-viscosity grade HPMC (hydroxypropyl methylcellulose) simultaneously improved the elasticity and durability of OGs, protecting them from mechanical and enzymatic degradation. The absorption of the curcumin fraction remaining in the gastrointestinal loop of rats was mainly influenced by the curcumin form in the OGs. In particular, the NLC significantly enhanced the intestinal absorption of curcumin after oral administration. These findings suggest that OGs based on Cur-NLCs act as an effective means to encapsulate curcumin and deliver it to the target site, achieving high levels of intestinal absorption. O’Sullivan et al. (2017) [23] investigated the digestion of β-carotene-fortified OGs structured with ethylcellulose and found that the structured oil interfered with lipid hydrolysis by inhibiting potential lipase activity. This prolonged the retention time of β-carotene in the gastrointestinal tract, resulting in a more consistent absorption pattern over time without fluctuations in plasma concentrations. Turmeric extract (TE), considered a great bioactive compound, has shown many health benefits, such as anticancer and anti-inflammatory effects [24]. Therefore, the release and digestion profiles of TE-incorporated OG (TE-OG) were investigated using in vitro gastrointestinal conditions.

This study addresses a significant gap in the field of oleogelation by addressing the substantial decline in oil-binding capacity and overall stability of oleogels produced via direct methods over time. Due to limitations with direct methods, such as the presence of a certain amount of saturated fat and an undesirable waxy taste, studies have been conducted on alternative approaches to improve oleogel properties. The indirect method using an emulsion template can improve oil-binding capacity, ensuring a more efficient and stable oleogel structure [25,26]. This optimization not only corrects the previous shortcomings but also improves the overall rheological properties of the oleogels, providing a more resilient and stable product [27].

The main objectives of this study are to prepare emulsion-templated TE-OG as an alternative to solid fats and to improve their oxidative stability and rheological properties through the combinations of XG and SL as oleogelators. The prepared TE-OG was characterized by their oil-binding capacity (OBC), oxidative stability, release profile of TE, and lipid digestion. The optimized TE-OG was used as an alternative to butter to prepare pound cakes and their rheological properties were compared to those of the control pound cake prepared using commercial butter.

## 2. Results and Discussion

### 2.1. Characterization of TE Emulsion

#### 2.1.1. Characterization of Emulsion with Different XG and SL Concentrations

**Figure 1:**
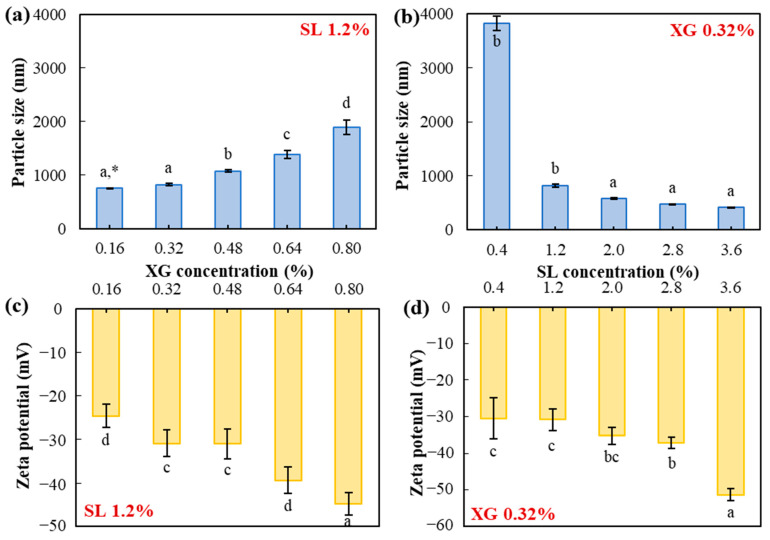
Particle size (**a**,**b**) and zeta potential (**c**,**d**) of emulsion with different XG (**a**,**c**) and SL (**b**,**d**) concentrations. * Different letters in each graph indicate a significant difference at *p* < 0.05.

Figure 1a presents the effect of different XG concentrations (0.16, 0.32, 0.48, 0.64, and 0.80%) on the particle size of the emulsion containing 1.2% SL. The particle size of TE emulsion significantly (*p* < 0.05) increased from 753.10 ± 3.70 nm to 1890.67 ± 140.38 nm with increasing XG concentrations in the aqueous phase with the formation of the outer layer of the particles. At higher concentrations, XG creates a dense, gel-like matrix when it interacts with liquids, entangling molecules and trapping water, leading to particle expansion. The increasing concentration of XG led to the formation of a more extensive molecular network, resulting in an enlargement of particle size. This phenomenon can be attributed to the moisture retention and thickening properties inherent in XG, aligning with commonly observed trends associated with its utilization as a thickening agent or gelling agent [28]. As the concentration of xanthan gum (XG) increases, the polymer molecules have a greater tendency to interact with the liquid and form a dense gel-like matrix [29]. These interactions involve the entanglement of XG molecules, creating a network that effectively traps water molecules, and contributes to the particle expansion [30]. The observed concentration-dependent formation of a more extensive molecular network is due to the increasing density of polymer interactions.

Conversely, the zeta potential was significantly (*p* < 0.05) decreased from −24.59 ± 2.64 mV to −44.79 ± 2.59 mV with increasing XG concentration (Figure 1c). This decline in zeta potential can be elucidated by the stabilizing effect of XG present on the outer layer of the emulsion [31]. The incorporation of negatively charged functional groups, such as carboxyl groups, by XG contributed to generating a negative zeta potential [32]. In general, an emulsion is considered stable if the absolute value of the zeta potential exceeds 30 mV [24]. The optimal amount of XG was 0.32%, allowing for the maintenance of a small particle size (824.73 ± 27.67 nm) and a stable zeta potential (−30.87 ± 3.06 mV) with the minimum quantity of XG.

Figure 1b illustrates the variations in particle size observed in the emulsion with a fixed XG concentration of 0.32%, while varying the SL concentration (0.4, 1.2, 2.0, 2.8, and 3.6%). The particle size decreased significantly (*p* < 0.05) from 3821.23 ± 133.51 nm to 413.20 ± 5.28 nm with the SL concentration as an emulsifier. This phenomenon aligns with the findings of Park et al.’s (2018) [33] research, indicating a uniform formation of particle size in the encapsulation system, with a decrease observed as the emulsifier concentration increased. Sufficient amounts of surfactant are essential to produce small and uniform particles. Furthermore, the zeta potential experienced a significant (*p* < 0.05) decrease from −30.44 ± 5.65 mV to −51.34 ± 1.67 mV with increasing SL concentration (Figure 1d). The elevation of the negative charge in the emulsion can occur due to the anionic nature of phospholipids present in SL and the adsorption of OH^−^ species at the oil–water interface. In all SL concentrations, the zeta potential exhibited stability, effectively preventing particle aggregation. Consequently, 0.32% XG and 1.2% SL was selected as the optimal condition of emulsion preparation, showing the dual advantages of ensuring stability and maintaining a consistent particle size.

#### 2.1.2. Characterization of Emulsion with Different TE Concentrations

**Figure 2:**
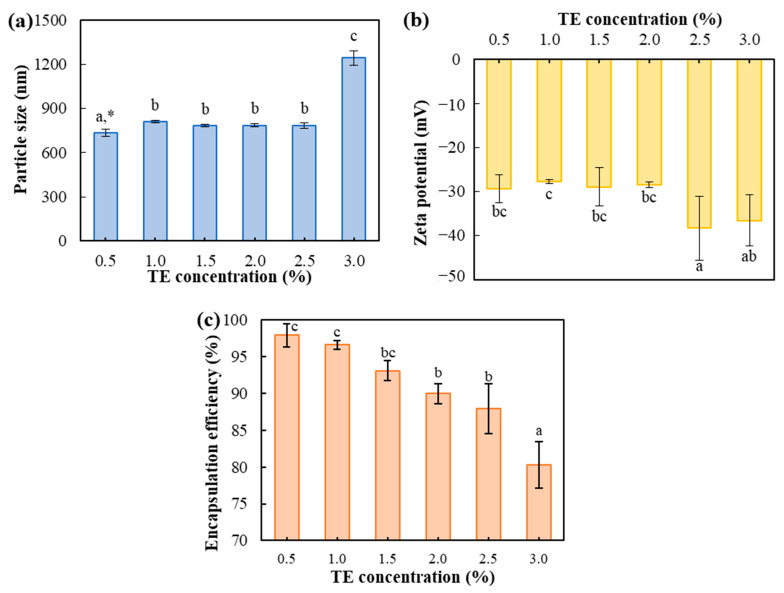
Particle size (**a**), zeta potential (**b**), and encapsulation efficiency (**c**) of TE emulsion (XG 0.32%/SL 1.2%) with different TE concentrations. * Different letters in each graph indicate a significant difference at *p* < 0.05.

To enhance functionality, varying concentrations of TE (0.5, 1.0, 1.5, 2.0, 2.5, and 3.0%) were incorporated into the optimized TE emulsion containing 0.3% XG and 1.2% SL. The complex mixture of compounds present in TE makes accurate quantitative analysis difficult. Therefore, the encapsulation efficiency (EE) was measured using curcumin, the primary bioactive constituent of TE. As shown in Figure 2a, the particle size increased from 734.33 ± 24.39 nm to 1242.77 ± 49.74 nm with increasing TE concentration. Additionally, the zeta potential was found to decrease slightly, ranging from −29.39 ± 3.20 mV to −36.60 ± 5.79 mV, as shown in Figure 2b. It was noted that the addition of 2% TE was found to have no significant effect on both particle size and zeta potential of TE emulsion.

The EE of curcumin in the TE emulsion is shown in Figure 2c. The addition of up to 1% TE resulted in a high EE of above 95%, ranging from 96.62 ± 0.56% to 97.93 ± 1.61%. On the other hand, TE concentrations ranging from 1.5% to 3.0% resulted in a significant (*p* < 0.05) and notable decrease in EE ranging from 93.08 ± 1.34% to 80.26 ± 3.15%. Therefore, we selected the optimal TE concentration as 1.0%, which demonstrated outstanding EE while maintaining a small particle size and stable zeta potential value.

### 2.2. Characterization and Optimization of TE-OG

#### 2.2.1. Oil-Binding Capacity (%)

The production of emulsion-templated OGs requires a sufficient amount of oleogelator to show the strengthening of the interfacial network [34]. Since optimal emulsion conditions determined based only on particle size and zeta potential may not be suitable for OG formation, TE-OG preparation was performed using all XG and SL concentrations tested in Section 2.1. This step helped characterize the TE-OG and assess their suitability for the intended application.

Table 1a shows the OBC values for different XG concentrations, which indicate the ability of the three-dimensional network to retain SO within the TE-OG. TE-OG formulated at XG concentration above 0.32% exhibited OBC values exceeding 99.99% during 120 h of storage. In contrast, TE-OG prepared with 0.16% XG concentration showed a significant (*p* < 0.05) decrease in OBC from 16.027 ± 1.555% to 7.823 ± 0.586% during the same storage. It is known that preserving oil droplets within well-formed emulsions through freeze-drying leads to the formation of dense, tightly packed structures [16]. However, the TE-OG prepared at 0.16% XG leads to the rupture and coalescence of the surrounding wall of the oil droplets due to the insufficient XG at the interface of oil droplets [14].

Table 1b presents the effect of different SL concentrations on the OBC value of TE-OG containing an XG concentration of 0.32%. TE-OG formulated at SL concentrations above 1.2% exhibited OBC values above 99.99% over 120 h storage. In contrast, TE-OG formulated at 0.4% SL concentration showed a significant (*p* < 0.05) decrease in OBC from 99.999 ± 0.005% to 92.831 ± 0.444% during the same storage period. The decline in OBC presents a potential challenge to TE-OG in maintaining stable SO reserves within the structure over time [35]. This instability can cause problems such as oil separation or migration, which are generally undesirable properties in various food and product applications.

#### 2.2.2. Rheological Property

**Figure 3:**
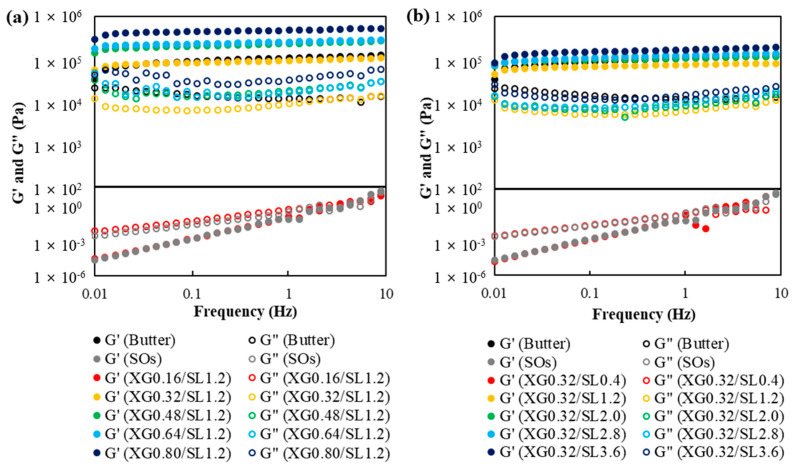
Frequency sweeps of TE-OGs with different XG (**a**) and SL (**b**) concentrations.

The rheological properties of TE-OG with various XG and SL concentrations were investigated through frequency sweeps as illustrated in Figure 3a and Figure 3b, respectively. Storage modulus (G′) and loss modulus (G″) were investigated as they represent the elastic and viscous properties of the samples, respectively. With increasing XG concentration, both G′ and G″ of the TE-OG showed an upward trend, indicating the basis of gels characterized by improved elasticity and viscosity. This increase in both moduli is attributed to the potential of XG to build stronger and more organized networks in TE-OG. Increasing XG concentration leads to an increased availability of XG molecules for interaction, resulting in a dense molecular matrix within TE-OG [36]. This matrix plays a crucial role in enhancing the mechanical properties of TE-OG, leading to a gel-like structure with improved G′ and G″ values, an observation consistent with previous findings on mixtures of rice starch and xanthan gum [37]. Interestingly, TE-OG formulated with XG concentrations above 0.32% showed a higher G′ value over G″ value, whereas those formulated with a 0.16% XG concentration displayed a higher G″ value over G′ value. A similar trend was observed in G′ and G″ of TE-OG with increasing SL concentrations. TE-OG prepared at 0.4% SL concentration showed a higher G″ value than G′ value. In contrast, TE-OG formulated at SL concentrations above 1.2% showed higher G′ values than G″ values. The presence of SL, acting as a surfactant, can increase both G′ and G″ of TE-OG [38]. Additionally, the interaction between the polymer and the surfactant strengthened the physical crosslinking and made the polymer network more robust [39].

It is widely recognized that G″ values smaller than G′ values lead to solid-like behavior in gel systems [40]. These characteristics hold significance in various food processing applications wherein maintaining structural integrity and shape is important, similar to the requirements observed for commercial butter. Therefore, TE-OG exhibiting elevated G′ values are more appropriate to emulate the attributes of commercial butter and prove effective in applications requiring a solid-like behavior. As a result, an XG concentration of 0.3%, SL concentration of 1.2%, and TE concentration of 1.0% were selected as the optimal conditions for TE-OG.

### 2.3. Oxidative Stability

For the accelerated assessment of oxidative stability, the Schaal oven test [41] was employed, subjecting the samples to accelerated oxidation at 60 °C. This accelerated oxidation evaluation method can simulate sample exposure to 60 °C for one day and storage at one month of ambient storage. This test is designed to estimate the behavior of samples over a long period under typical storage conditions and to evaluate the shelf life and stability of the product. As a result, TE-OGs were stored in a light-proof oven at 60 °C for 12 days. In this study, the peroxide value (PV), ρ-anisidine value (ρ-AV), and total oxidation value (TOTOX) of the TE-OG were evaluated for 12 days at 60 °C. PV and ρ-AV serve as indicators of primary and secondary oxidation product formation, respectively [41]. For comparison, a control OG was prepared using the same manufacturing procedure as the TE-OG, except for TE incorporation. Additionally, TE-SO samples were prepared by dissolving TE in SO, and commercially available butter and soybean oil (SO) were prepared as control samples.

As shown in Table 2a, SO showed a rapid increase in PV, reaching 82.319 ± 5.163 mmol/kg after 10 days, while TE-SO reached 17.494 ± 0.675 mmol/kg after 12 days. Similarly, OG showed 39.082 ± 0.963 mmol/kg in PV after 12 days, while TE-OG reached only 2.953 ± 0.439 mmol/kg after 12 days. These observed effects may be due to the antioxidant properties of curcumin or other bioactive compounds in TE [24]. Curcumin is a very powerful lipophilic antioxidant known for its ability to effectively scavenge various reactive oxygen species, including hydroxyl radicals and nitrogen dioxide radicals [42]. As shown in Table 2b, the gradual increase in ρ-AV of SO from 10.605 ± 0.388 to 49.888 ± 0.913 was alleviated by incorporating TE (from 10.272 ± 1.815 to 16.703 ± 0.175). Remarkably, its entrapment within the OG structure formed by XG and SL, reducing air interaction and retarding oxidation, appears to confer antioxidant activity similar to that observed with TE-SO. In contrast, butter maintained the lowest PV and ρ-AV values, ranging from 0.296 ± 0.293 mmol/kg to 2.292 ± 0.229 mmol/kg and from 0.731 ± 0.124 to 3.298 ± 0.669, respectively.

Table 2c displays TOTOX results, serving as a holistic metric for assessing the overall quality of the oil, derived from the combination of PV and ρ-AV values. Because SO has a high polyunsaturated fatty acids (PUFAs) content, it has much higher TOTOX values, making it highly susceptible to oxidative degradation. OG samples (from 18.762 ± 1.250 to 92.666 ± 2.068) had significantly (*p* < 0.05) lower TOTOX values compared to SO (from 18.636 ± 0.253 to 183.519 ± 3.228), indicating superior oil quality due to reduced primary and secondary oxidation products. It was observed that OG can significantly (*p* < 0.05) improve the oxidative stability of SO by almost double, even in the absence of natural antioxidants. Additionally, the addition of TE increased the oxidative stability of OG by approximately 4.5-fold, and the TOTOX values of TE-OG samples ranged from 12.295 ± 0.692 to 18.986 ± 2.298. Commercially available butter showed the lowest oxidation stability due to the absence of double bonds in vulnerable positions within its molecular structure [43]. Other research supports this by suggesting that oleogelation improves the oxidative stability of oils, akin to adding antioxidants [41]. Reports by Su et al. (2023) [44] and Zhuang et al. (2021) [45] highlight how emulsion-templated OGs, structured with different materials, improve oxidative stability by preventing oil droplets from directly interacting with oxygen, leading to a significant delay in the oxidation process.

### 2.4. In Vitro Lipid Digestion

#### 2.4.1. Release of Curcumin and Free Fatty Acids

**Figure 4:**
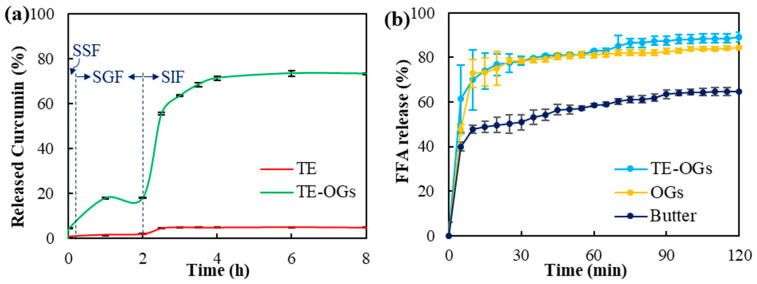
The released amount of curcumin (**a**) and free fatty acids (**b**) during the in vitro lipid digestion.

Release of curcumin from TE and TE-OG was evaluated after digestion in enzyme-free simulated salivary fluid (SSF), simulated gastric fluid (SGF), and simulated intestinal fluid (SIF) (Figure 4a). In SSF, minimal curcumin release was observed, with TE showing 0.90% of curcumin release, while TE-OG showed 4.66% of curcumin release. After SGF incubation, the release of curcumin increased to 2.01% and 18.07% in TE and TE-OG, respectively. The relatively high release amount of curcumin from TE-OG suggests that its solubility is improved due to encapsulation [46]. However, its release was less than 20%, which indicates the stable maintenance of encapsulated curcumin under acidic gastric conditions [47]. Conversely, a significant release of encapsulated curcumin occurred after incubation in SIF, and 73.22% of curcumin was released in TE-OG after digestion. This increased release appears to promote the dissolution of curcumin due to the formation of mixed micelles [48]. However, TE released only 4.98% of curcumin after digestion in SIF, which may be due to its low solubility and potential degradation in the SIF environment, as curcumin is known to be unstable in neutral-to-alkaline conditions [24]. These findings are consistent with previous reports describing significantly greater curcumin release using the encapsulated system compared to the unencapsulated one [49]. Additionally, curcumin encapsulated in XG was reported to exhibit a higher release of both SGF and SIF in comparison with neat curcumin in a previous study [50]. The incorporation of SL into TE-OG was demonstrated to augment the curcumin’s solubility through its interaction with the interface of oil droplets [51].

The lipid digestion profiles were closely examined by monitoring the release of free fatty acids (FFAs) from TE-OG, OG, and butter throughout in vitro digestion. As shown in Figure 4b, the release of FFAs shows a sharp surge in the initial minutes of digestion, followed by a gradual increase. Notably, both TE-OG and OG had higher FFA releases compared to butter. TE-OG and OG released 89.16 ± 2.12% and 84.29 ± 0.77% of FFAs, respectively, whereas butter released 64.66 ± 1.65% of FFAs after 120 min digestion. This increased release of TE-OG and OG may be due to the larger surface area provided by lipid droplets, which makes them more susceptible to enzymatic hydrolysis than butter [19]. Additionally, the amphiphilic nature of XG molecules could potentially contribute to the formation of mixed micelles during digestion, thereby improving the solubility of FFAs [52]. These findings specifically suggest that oleogelation with XG has a positive effect on the digestion of triacylglycerols found in SO.

#### 2.4.2. Microstructural Analysis

**Figure 5:**
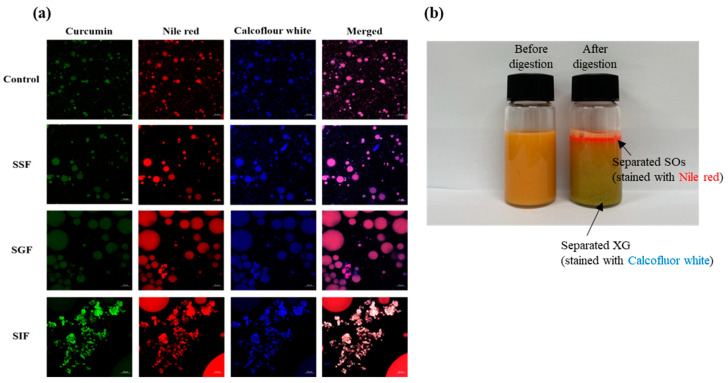
Confocal laser scanning microscope (CLSM) (**a**) and visual appearance images (**b**) of TE-OG samples before and after in vitro lipid digestion.

Microstructural analysis during in vitro digestion shows the evolution of TE-OGs (Figure 5a). Upon staining with fluorescent dyes Nile red and Calcofluor white, the initial observation unveiled the presence of spherical particles attributed to the emulsion template. Curcumin, with its intrinsic fluorescence, can be detected at 425/480 nm (excitation/emission) without dye. Under SGF, stable spherical particles were observed with slight separation of the XG layer. Subsequent digestion in SIF resulted in an amorphous appearance, indicative of lipid degradation and droplet aggregation due to the lipase and bile salt actions. This is visually confirmed by comparing samples containing dyed SO (Nile red) and XG (Calcofluor white) before and after digestion in Figure 5b to confirm that precipitation of the XG has occurred (blue color). These findings align with the alterations in microstructure observed throughout the in vitro digestion, as depicted in Figure 5a.

### 2.5. The Applicability of TE-OG to Pound Cake

#### 2.5.1. Texture Profile Analysis (TPA)

The pound cakes produced were classified as PC-0, PC-20, PC-40, PC-60, PC-80, and PC-100, depending on the content of TE-OG, from 0% to 100%, respectively. As summarized in Table 3, the addition of 20 to 100% of TE-OG content was found to have a positive effect on the hardness and chewiness of the pound cakes compared to PC-0. Hardness, which refers to the force required for a specific deformation of a food product [53], has been shown to increase when incorporating TE-OG as an alternative to butter. This increase in hardness means a harder and more resistant texture, which requires increased effort during mastication and chewing, affecting the sensory experience of consuming the pound cake. Chewiness, which pertains to the tactile sensation in the mouth characterized by prolonged, resilient resistance during mastication originating from the food, also increased with TE-OG replacement. Increased hardness and chewiness, requiring more force during mastication, have been reported to have a potential negative impact on the overall palatability of foods [14].

#### 2.5.2. Micro-Computed Tomography (micro-CT)

**Figure 6:**
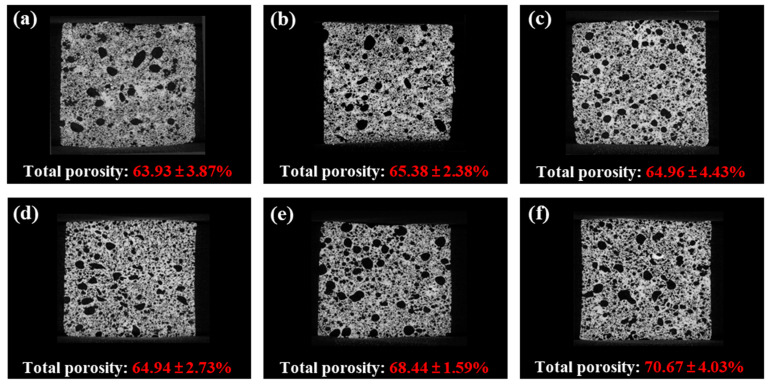
Micro-computed tomography (micro-CT) images and total porosity (%) of PC-0 (**a**), PC-20 (**b**), PC-40 (**c**), PC-60 (**d**), PC-80 (**e**), and PC-100 (**f**).

Micro-computed tomography (micro-CT) was employed to assess the microstructural characteristics and porosity of the pound cake. Within micro-CT images, the pores within the sample manifest as dark regions, with their intensity increasing proportionally to the density attributed to X-ray scattering [11]. As depicted in Figure 6, the micro-CT images of samples with incorporated butter display regions of increased brightness. As the amount of TE-OG increased, these bright areas decreased, and a distribution of small pores appeared throughout the cake. The observed increase in porosity was statistically significant (*p* < 0.05), increasing from 63.93 ± 3.87% for PC-0 to 70.67 ± 4.03% for PC-100. An augmentation in porosity resulting from the substitution with TE-OG can be ascribed to the presence of emulsifiers within TE-OG [54]. These emulsifiers facilitate the formation of minute and uniformly dispersed pores within the sample, thereby enhancing the overall porosity. Ye et al. (2019) [55] reported a similar phenomenon, where they employed ethylcellulose oleogel in bread formulation, resulting in enhanced porosity compared to bread incorporating conventional solid fat.

Designed to structure oils, the unique properties of TE-OG play a pivotal role in entrapping a multitude of air bubbles in the matrix of the sample. This organizational configuration not only enhances the amalgamation and steadying of smaller bubbles but also yields an end product with pores uniformly dispersed [56]. These distinctive characteristics significantly contribute to the development of a cake with a tender texture, as it diminishes hardness and chewiness, contrasting with cakes incorporating butter. Furthermore, as indicated in Table 3, the enduring springiness observed in the PC-100 sample can be attributed to the substantial elastic modulus and loss modulus demonstrated by TE-OG (refer to Figure 3). This sustained springiness highlights the stability of TE-OG, maintaining the structural integrity and textural excellence of the pound cake over an extended duration.

## 3. Conclusions

Turmeric extract-incorporated oleogels (TE-OGs) formulated with xanthan gum (XG) and soy lecithin (SL) demonstrated high oil-binding capacity (OBC), favorable rheological properties, and desirable oxidative stability. In vitro digestion experiments revealed that TE-OG effectively protected curcumin from gastric degradation and facilitated its release during intestinal digestion. When used as a solid fat substitute in pound cake formulation, the optimized TE-OG resulted in a softer texture, reduced hardness of the pound cake, and increased overall porosity. Notably, the pound cakes made with TE-OG exhibited smaller and more evenly distributed air bubbles, aligning with the growing consumer preference for a softer and more uniform texture. These findings underscore the viability and effectiveness of TE-OGs as a substitute for butter, enabling the production of high-quality bakery products with improved textural attributes. This study presents a promising approach to enhance functionality by incorporating functional materials into oleogels, suggesting the potential for further exploration and research in this direction.

## 4. Materials and Methods

### 4.1. Materials

SO and TE powder were obtained from Ottogi Corp., Ltd. (Anyang, Republic of Korea). XG with a viscosity ranging from 1200 to 1600 cPs in a 1% solution in 1% KCl was purchased from Deosen Biochemical (Ordos) Ltd. (Ordos, Inner Mongolia, China). SL was acquired from Solae LLC. (St. Louis, MO, USA). ρ-Anisidine was purchased from Tokyo Chemical Industry Co., Ltd. (Nihonbashi-honcho, Chuo-ku, Tokyo, Japan). Sodium thiosulfate, Nile red, and Calcofluor white dyes were purchased from Sigma-Aldrich (St. Louis, MO, USA). Isooctane, acetic acid, and potassium iodide (KI) were obtained from Daejung Chemicals (Seoul, Republic of Korea). Commercial butter (Anchor butter) was purchased from Fonterra Cooperative Group Ltd. (Auckland, New Zealand). Whole egg liquid and non-fat dry milk were purchased from KC Feed Co., Ltd. (Yeongcheon, Republic of Korea) and Seoul Milk Co. (Seoul, Republic of Korea), respectively. Sugar and flour were purchased from CJ CheilJedang Co., Ltd. (Seoul, Republic of Korea).

### 4.2. Preparation of TE-OGs

To produce TE-OGs, an emulsion served as the template. Initially, XG was solubilized in distilled water to generate an aqueous phase. The oil phase was formulated by dissolving TE powder and SL in SO. Aqueous and oil phases were combined in a 6:4 ratio, creating a coarse emulsion, followed by probe-type ultrasonication for 5 min at 20 kHz and 40% amplitude. Subsequently, the mixture underwent freeze-drying for 72 h to produce TE-OGs. The produced TE-OGs were carefully transferred into Falcon tubes, hermetically sealed and subsequently stored at −20 °C for preservation.

### 4.3. Encapsulation Efficiency (EE)

The EE of the TE-OG was assessed through high-performance liquid chromatography (HPLC) analysis. The dispersion of TE-OG was assessed by it being subjected to stirring in hexane at 25 °C for 2 h and then centrifuged at 10,000 rpm for 30 min. The resulting supernatant was combined with methanol in a 1:9 (*v*/*v*) ratio and subjected to HPLC analysis using a Shimadzu D-20A system (Kyoto, Japan) equipped with a UV absorbance detector. A ZORBAX Extend-C18 column (4.6 × 250 mm, 5 µm; Agilent Technologies, Santa Clara, CA, USA) was employed to quantify the curcumin content. The mobile phase consisted of acetonitrile and 2% acetic acid (65:35, *v*/*v*), pumped at a flow rate of 1 mL/min. An injection volume of 20 μL was utilized, and detection occurred at 425 nm. The EE was computed using the following Equation (1):

EE (%) = [(*C_i_* − *C_s_*)/*C_i_*] × 100 (1)

where *C_i_* is the initial amount of curcumin in the TE-OG before the centrifuge, and *C_s_* is the amount of free curcumin in the supernatant after the centrifuge.

### 4.4. Oil-binding Capacity (OBC)

The OBC of TE-OG samples was assessed using the centrifuge method [57]. Initially, the prepared TE-OG samples were transferred to centrifuge tubes and equilibrated at 25 °C and 50% RH for 3 h before analysis. Subsequently, the tubes were centrifuged at 10,000 rpm at 4 °C for 15 min. After centrifugation, the tubes were inverted onto filter paper to drain excess oil, and the mass of the tubes was recorded over time. OBC was determined through the application of the following Equation (2):

(2) $$OBC\left(\%\right)=\left(\frac{m_{3}-m_{1}}{m_{2}-m_{1}}\right)\times 100$$

where *m*_1_ represents the mass of the empty tube, *m*_2_ corresponds to the combined mass of the tube and sample before centrifugation, and *m*_3_ represents the mass of the tube and sample following the centrifugation and removal of excess oils.

### 4.5. Oxidative Stability

PV and ρ-AV were conducted following the AOCS official methods (CD 8b-90 and CD 18-90, respectively). The TOTOX value, serving as an indicator of the initial deterioration in the oil, offers comprehensive insights into both primary and secondary oxidation products and was calculated as the following Equation (3):

(3) TOTOX=2PV+ρ−AV

### 4.6. In Vitro Digestion

The simulation of the digestion process, encompassing oral, gastric, and small intestine phases, was executed according to Hong et al. (2024) [58]. During the oral simulation, 0.5 g of TE-OG was combined with 9.5 g of SSF, and the pH was set to 7 with 0.1 N NaOH. The mixture was incubated at 37 °C with agitation for 2 min. In the gastric simulation, 10 g oral bolus was mixed with an equivalent amount of SGF, followed by the addition of pepsin and gastric lipase. The pH was lowered to 2 using 0.1 N HCl, and the resultant mixture was incubated at 37 °C for 2 h. During the intestinal simulation, a mixture was prepared by combining 20 g of gastric chyme with an equivalent amount of SIF and introducing pancreatin, pancreatic lipase, and bile salt.

#### 4.6.1. Free Fatty Acids (FFAs) Release

During the intestinal digestion, the released amount of FFAs from the TE-OG was titrated with a pH-stat (907 Titrando, Metrohm, Herisau, Switzerland) at 37 °C for 2 h. The quantity of 0.1 N NaOH utilized for pH adjustment to 7 serves as a measure for calculating the amount of released FFA as in the following Equation (4):

(4) $$FFA\;released(\%)=\frac{V_{NaOH}\times M_{NaOH}\times M_{Lipid}}{W_{Lipid}\times 2}\times 100$$

where *V_NaOH_* represents the volume of the utilized NaOH (L), *M_NaOH_* indicates the concentration of the used NaOH (0.1 N), *M_Lipid_* stands for the molecular weight of the lipid (g/mol), and *W_Lipid_* denotes the total mass of the lipid employed in the intestinal digestion stage (g).

#### 4.6.2. Curcumin Release from TE-OG

The curcumin release from TE-OG was assessed following the methodology outlined in prior studies [49]. The TE-OG samples were combined with enzyme-free SSF, SGF, and SIF at 37 °C for 0, 1, 2, 2.5, 3, 4, 6, and 8 h. In each digesta, an HPLC was used to determine the amount of curcumin as described in Section 4.3.

#### 4.6.3. Confocal Laser Scanning Microscopy (CLSM)

For the examination of morphological changes in TE-OG samples before and after digestion, a Confocal Laser Scanning Microscope (LSM 710, Zeiss, Gottingen, Germany) was employed. SO and XG were pre-reacted with Nile red (0.02% *w*/*v*) and Calcofluor white (1% *w*/*v*), respectively, before their incorporation into the emulsion and OG. Monitoring alterations in the lipid phase were achieved using Nile red, while Calcofluor white was employed to visualize the alterations in the morphology of XG during the digestion of TE-OG. The excitation wavelengths for Nile red and Calcofluor white were 515–530 nm and 440 nm, respectively, while their emission wavelengths were 525–605 nm and 500–520 nm, correspondingly. 

### 4.7. Applicability to Pound Cake

#### 4.7.1. Preparation of Pound Cake

To assess the feasibility of TE-OG as a substitute for a solid fat, pound cakes were formulated by replacing butter with TE-OG at different proportions (0, 20, 40, 60, 80, and 100%). Initially, 300 g of fats and 300 g of sugar were mixed. Subsequently, 300 g of whole egg, 300 g of wheat flour, 32 g of non-fat dry milk, 13 g of baking powder, and 2 g of salt were introduced. Finally, a total of 200 g of water was introduced and mixed, after which the batter was scraped off. The resulting batters were placed into pans and baked in an oven with upper and bottom temperatures set at 185 °C and 180 °C, respectively, for 30 min.

#### 4.7.2. Texture Profile Analysis (TPA)

The textural characteristics of the fabricated pound cakes were evaluated by employing a Universal Testing Machine (UTM; Z010TN, Zwick GmbH & Co. KG, Ulm, Germany) [58]. Initially, 2 × 2 × 2 cm-sized samples of the fabricated pound cake taken from the cake’s central region were positioned on a flat plate. Subsequently, a 3.5 cm diameter probe compressed the sample to 50% of its original height at a rate of 10 mm/min, whereas both pre-speed and post-speed were set at 50 mm/min. All assessments were conducted at ambient temperature, and each measurement was repeated at least 5 times for accuracy and consistency.

#### 4.7.3. X-ray Micro-Computed Tomography (CT) Analysis

The internal structure of the produced pound cakes was non-destructively visualized using an X-ray micro-CT system (XT H 225, Nikon Corp., Tokyo, Japan). Initially, samples of the fabricated pound cake, measuring 4 cm (width) × 4 cm (height) × 3 cm (depth), were taken from the central region of the cakes and positioned. These samples were affixed to a rotating stage within the Micro-CT apparatus and subjected to scanning conditions with a voltage of 100 kV and a current of 180 µA. Utilizing myVGL 3.0 software (Volume Graphics GmbH, Heidelberg, Germany), the images were stacked to generate 3D representations, with pore spaces delineated as black regions.

### 4.8. Statistical Analysis

The data are reported in triplicate for each measurement, presented as mean values along with their corresponding standard deviations (mean ± SD). Statistical analyses were carried out according to a completely randomized design with three independent experiment replications using one-way analysis of variance (ANOVA) with SPSS software (SPSS Inc., Chicago, IL, USA). To discern variations among the means, Duncan’s multiple range test (*p* < 0.05) was utilized for statistical analysis.

## Figures and Tables

**Table 1:** Oil-binding capacity (%) of TE-OG with different XG (a) and SL (b) concentrations by storage times.

(a)					
Time (h)	XG-0.16/SL-1.2	XG-0.32/SL-1.2	XG-0.48/SL-1.2	XG-0.64/SL-1.2	XG-0.80/SL-1.2
1	16.027 ± 1.555 ^d,A,*^	99.999 ± 0.000 ^a,B^	100.000 ± 0.002 ^a,B^	99.994 ± 0.015 ^a,B^	100.000 ± 0.002 ^b,B^
3	14.271 ± 1.509 ^c,A^	99.999 ± 0.000 ^a,B^	99.999 ± 0.001 ^a,B^	99.991 ± 0.013 ^a,B^	100.000 ± 0.001 ^b,B^
6	10.040 ± 0.488 ^b,A^	99.997 ± 0.005 ^a,B^	99.999 ± 0.001 ^a,B^	99.992 ± 0.012 ^a,B^	100.000 ± 0.000 ^b,B^
12	9.580 ± 0.168 ^b,A^	99.998 ± 0.001 ^a,B^	99.999 ± 0.001 ^a,B^	99.991 ± 0.012 ^a,B^	99.999 ± 0.001 ^ab,B^
24	9.040 ± 0.484 ^ab,A^	99.998 ± 0.001 ^a,B^	99.999 ± 0.001 ^a,B^	99.991 ± 0.012 ^a,B^	99.999 ± 0.001 ^ab,B^
72	7.877 ± 0.551 ^a,A^	99.996 ± 0.000 ^a,B^	99.998 ± 0.000 ^a,B^	99.990 ± 0.013 ^a,B^	99.999 ± 0.001 ^ab,B^
120	7.823 ± 0.586 ^a,A^	99.998 ± 0.001 ^a,B^	99.999 ± 0.002 ^a,B^	99.990 ± 0.013 ^a,B^	99.998 ± 0.001 ^a,B^
** (b) **					
**Time (h)**	**XG-0.32/SL-0.4**	**XG-0.32/SL-1.2**	**XG-0.32/SL-2.0**	**XG-0.32/SL-2.8**	**XG-0.32/SL-3.6**
1	99.999 ± 0.005 ^e,A^	99.999 ± 0.000 ^a,A^	100.001 ± 0.001 ^b,A^	99.999 ± 0.003 ^b,A^	99.999 ± 0.001 ^a,A^
3	98.876 ± 0.733 ^d,A^	99.999 ± 0.000 ^a,B^	99.990 ± 0.017 ^ab,B^	100.000 ± 0.002 ^b,B^	99.999 ± 0.002 ^a,B^
6	98.190 ± 0.533 ^d,A^	99.997 ± 0.005 ^a,B^	99.952 ± 0.025 ^a,B^	99.999 ± 0.001 ^ab,B^	99.998 ± 0.003 ^a,B^
12	97.173 ± 0.415 ^c,A^	99.998 ± 0.001 ^a,B^	99.954 ± 0.023 ^a,B^	99.999 ± 0.001 ^ab,B^	99.998 ± 0.003 ^a,B^
24	96.594 ± 0.595 ^c,A^	99.998 ± 0.001 ^a,B^	99.953 ± 0.023 ^a,B^	99.998 ± 0.001 ^ab,B^	99.998 ± 0.003 ^a,B^
72	94.037 ± 0.264 ^b,A^	99.996 ± 0.000 ^a,B^	99.952 ± 0.024 ^a,B^	99.995 ± 0.001 ^a,B^	99.998 ± 0.000 ^a,B^
120	92.831 ± 0.444 ^a,A^	99.998 ± 0.001 ^a,B^	99.955 ± 0.023 ^a,B^	99.996 ± 0.000 ^ab,B^	100.000 ± 0.000 ^a,B^

* Different letters (a–e) and (A,B) indicate a significant difference at *p* < 0.05 in OBC at the same time and XG concentration, respectively.

**Table 2:** Changes in peroxide value (a), ρ-anisidine value (b), and total oxidation (TOTOX) value (c) of 12 days’ Schaal oven test.

(a)					
Time (days)	Peroxide value (mmol/kg)				
	Butter	SO	TE-SO	OG	TE-OG
0	0.296 ± 0.293 ^a,A,*^	4.016 ± 0.285 ^a,C^	1.312 ± 0.291 ^a,B^	3.846 ± 0.789 ^ab,C^	1.646 ± 0.507 ^ab,B^
2	0.455 ± 0.292 ^a,A^	3.995 ± 0.265 ^a,D^	1.800 ± 0.260 ^b,B^	3.269 ± 0.371 ^a,C^	1.423 ± 0.253 ^a,B^
4	0.294 ± 0.293 ^a,A^	20.936 ± 1.275 ^b,E^	2.990 ± 0.291 ^c,C^	5.363 ± 0.292 ^b,D^	1.637 ± 0.101 ^ab,B^
6	0.954 ± 0.265 ^b,A^	40.391 ± 1.826 ^c,D^	4.260 ± 0.102 ^d,B^	7.617 ± 0.244 ^c,C^	2.063 ± 0.085 ^bc,A^
8	0.813 ± 0.289 ^b,A^	69.007 ± 0.619 ^d,E^	5.688 ± 0.100 ^d,C^	17.282 ± 0.528 ^d,D^	2.041 ± 0.234 ^bc,B^
10	2.292 ± 0.229 ^d,A^	82.319 ± 5.163 ^f,D^	11.341 ± 0.081 ^e,B^	28.461 ± 1.341 ^e,C^	2.088 ± 0.082 ^c,A^
12	0.953 ± 0.065 ^c,A^	66.815 ± 1.263 ^e,E^	17.494 ± 0.675 ^f,C^	39.082 ± 0.963 ^f,D^	2.953 ± 0.439 ^d,B^
** (b) **					
**Time** **(days)**	**ρ-Anisidine value**				
	**Butter**	**SO**	**TE-SO**	**OG**	**TE-OG**
0	0.731 ± 0.124 ^a,A^	10.605 ± 0.388 ^a,BC^	10.272 ± 1.815 ^a,BC^	11.070 ± 0.353 ^b,C^	9.003 ± 0.326 ^ab,B^
2	0.881 ± 0.499 ^a,A^	11.656 ± 0.639 ^a,CD^	12.673 ± 0.522 ^b,D^	11.146 ± 0.507 ^b,C^	8.460 ± 1.270 ^a,B^
4	0.803 ± 0.192 ^a,A^	14.383 ± 0.539 ^b,D^	12.678 ± 1.372 ^b,C^	9.918 ± 0.467 ^a,B^	9.107 ± 1.395 ^ab,B^
6	1.895 ± 0.725 ^b,A^	22.551 ± 0.644 ^c,D^	15.113 ± 0.364 ^c,C^	10.160 ± 0.556 ^a,B^	9.569 ± 0.463 ^ab,B^
8	1.393 ± 0.220 ^ab,A^	27.720 ± 1.294 ^d,D^	12.109 ± 1.186 ^b,C^	10.052 ± 0.248 ^a,B^	9.694 ± 0.067 ^ab,B^
10	2.013 ± 0.092 ^b,A^	37.743 ± 0.577 ^e,E^	15.225 ± 0.663 ^c,D^	12.486 ± 0.087 ^c,C^	11.089 ± 0.749 ^b,B^
12	3.298 ± 0.669 ^c,A^	49.888 ± 0.913 ^f,D^	16.703 ± 0.175 ^c,C^	14.502 ± 0.146 ^d,B^	13.081 ± 2.074 ^c,B^
** (c) **					
**Time** **(days)**	**TOTOX value**				
	**Butter**	**SO**	**TE-SO**	**OG**	**TE-OG**
0	1.323 ± 0.478 ^a,A^	18.636 ± 0.253 ^a,C^	12.895 ± 2.089 ^a,B^	18.762 ± 1.250 ^ab,C^	12.295 ± 0.692 ^ab,B^
2	1.791 ± 0.791 ^a,A^	19.646 ± 0.790 ^a,D^	16.272 ± 0.830 ^b,C^	17.684 ± 0.683 ^a,C^	11.306 ± 1.557 ^a,B^
4	1.392 ± 0.568 ^a,A^	56.255 ± 2.898 ^b,D^	18.657 ± 1.414 ^c,C^	20.643 ± 0.420 ^b,C^	12.381 ± 1.232 ^ab,B^
6	3.802 ± 0.220 ^b,A^	103.334 ± 3.764 ^c,D^	23.634 ± 0.567 ^d,C^	25.394 ± 0.733 ^c,C^	13.694 ± 0.519 ^bc,B^
8	3.018 ± 0.759 ^b,A^	165.735 ± 2.017 ^d,E^	23.486 ± 1.144 ^d,C^	44.616 ± 1.040 ^d,D^	13.776 ± 0.533 ^bc,B^
10	6.598 ± 0.417 ^d,A^	202.380 ± 10.635 ^f,D^	37.908 ± 0.789 ^e,B^	69.409 ± 2.745 ^e,C^	15.266 ± 0.913 ^c,A^
12	5.204 ± 0.596 ^c,A^	183.519 ± 3.228 ^e,E^	51.691 ± 1.468 ^f,C^	92.666 ± 2.068 ^f,D^	18.986 ± 2.298 ^d,B^

* Different letters (a–f) and (A–E) indicate a significant difference in OBC at the same time and XG concentration, respectively (*p* < 0.05).

**Table 3:** Changes in texture parameters of pound cakes with different contents of TE-OG added.

Day	Sample	Hardness (N)	Adhesiveness (mJ)	Springiness	Chewiness (N)	Cohesiveness
0	PC-0	10.08 ± 1.39 ^c,A,*^	0.42 ± 0.09 ^a,A^	0.71 ± 0.08 ^b,AB^	2.13 ± 0.57 ^b,A^	0.27 ± 0.04 ^a,B^
	PC-20	9.79 ± 0.78 ^bc,A^	0.33 ± 0.06 ^a,A^	0.70 ± 0.05 ^b,A^	1.78 ± 0.30 ^ab,A^	0.26 ± 0.03 ^a,C^
	PC-40	9.37 ± 0.38 ^bc,A^	0.45 ± 0.08 ^a,A^	0.76 ± 0.02 ^b,B^	1.95 ± 0.08 ^b,A^	0.27 ± 0.01 ^a,B^
	PC-60	9.60 ± 0.32 ^bc,A^	0.36 ± 0.14 ^a,A^	0.74 ± 0.05 ^b,C^	1.78 ± 0.42 ^ab,B^	0.25 ± 0.04 ^a,B^
	PC-80	8.79 ± 0.26 ^ab,A^	0.51 ± 0.07 ^a,A^	0.60 ± 0.13 ^a,A^	1.41 ± 0.36 ^a,A^	0.25 ± 0.08 ^a,B^
	PC-100	7.88 ± 0.68 ^a,A^	0.41 ± 0.18 ^a,A^	0.77 ± 0.02 ^b,A^	1.78 ± 0.17 ^ab,A^	0.29 ± 0.01 ^a,C^
3	PC-0	15.75 ± 0.67 ^bc,B^	0.38 ± 0.11 ^a,A^	0.74 ± 0.02 ^b,B^	2.50 ± 0.39 ^b,A^	0.18 ± 0.06 ^b,A^
	PC-20	15.75 ± 0.70 ^bc,B^	0.42 ± 0.10 ^ab,A^	0.65 ± 0.05 ^ab,A^	1.91 ± 0.26 ^abc,A^	0.21 ± 0.01 ^b,B^
	PC-40	15.90 ± 0.77 ^c,B^	0.43 ± 0.11 ^ab,A^	0.64 ± 0.04 ^ab,A^	1.49 ± 0.49 ^ab,A^	0.14 ± 0.04 ^a,A^
	PC-60	16.02 ± 0.50 ^c,B^	0.51 ± 0.12 ^ab,A^	0.61 ± 0.05 ^a,A^	1.35 ± 0.28 ^a,A^	0.14 ± 0.02 ^a,A^
	PC-80	15.07 ± 0.43 ^ab,B^	0.61 ± 0.20 ^b,A^	0.67 ± 0.07 ^cab,A^	2.04 ± 0.41 ^abc,B^	0.20 ± 0.03 ^b,B^
	PC-100	14.96 ± 0.53 ^a,B^	0.39 ± 0.17 ^a,A^	0.72 ± 0.12 ^ab,A^	2.21 ± 0.48 ^bc,AB^	0.20 ± 0.02 ^b,B^
7	PC-0	19.75 ± 0.78 ^bc,C^	0.49 ± 0.18 ^ab,A^	0.64 ± 0.05 ^a,A^	2.13 ± 0.55 ^ab,A^	0.17 ± 0.03 ^b,A^
	PC-20	19.87 ± 0.64 ^c,C^	0.63 ± 0.22 ^b,B^	0.62 ± 0.08 ^a,A^	1.82 ± 0.36 ^a,A^	0.16 ± 0.03 ^b,A^
	PC-40	19.77 ± 0.65 ^bc,C^	0.37 ± 0.07 ^a,A^	0.64 ± 0.07 ^a,A^	1.72 ± 0.39 ^a,A^	0.14 ± 0.02 ^ab,A^
	PC-60	20.14 ± 0.70 ^c,C^	0.42 ± 0.08 ^ab,A^	0.66 ± 0.03 ^ab,B^	1.68 ± 0.21 ^a,AB^	0.13 ± 0.01 ^a,A^
	PC-80	18.98 ± 0.50 ^ab,C^	0.40 ± 0.10 ^a,A^	0.67 ± 0.04 ^ab,A^	1.67 ± 0.27 ^a,AB^	0.13 ± 0.01 ^ab,A^
	PC-100	18.88 ± 0.58 ^a,C^	0.42 ± 0.10 ^ab,A^	0.73 ± 0.01 ^b,A^	2.40 ± 0.32 ^b,B^	0.17 ± 0.04 ^b,A^

* Different letters (a–c) and (A–C) indicate a significant difference at *p* < 0.05 in OBC at the same time and XG concentration, respectively.

## Data Availability

The data presented in this study are available on request from the corresponding author. The data are not publicly available due to ethical.
